# Noninvasive blood tests for fetal development predict gestational age and preterm delivery

**DOI:** 10.1126/science.aar3819

**Published:** 2018-06-08

**Authors:** Thuy T. M. Ngo, Mira N. Moufarrej, Marie-Louise H. Rasmussen, Joan Camunas-Soler, Wenying Pan, Jennifer Okamoto, Norma F. Neff, Keli Liu, Ronald J. Wong, Katheryne Downes, Robert Tibshirani, Gary M. Shaw, Line Skotte, David K. Stevenson, Joseph R. Biggio, Michal A. Elovitz, Mads Melbye, Stephen R. Quake

**Affiliations:** 1 Departments of Bioengineering and Applied Physics, Stanford University and Chan Zuckerberg Biohub, Stanford, CA 94305, USA; 2 Department of Epidemiology Research, Statens Serum Institute, Copenhagen 2300, Denmark; 3 Department of Statistics, Stanford University, Stanford, CA94305, USA; 4 Department of Pediatrics, Stanford University School of Medicine, Stanford, CA 94305, USA; 5 Maternal and Child Health Research Center, Department of Obstetrics and Gynecology, University of Pennsylvania School of Medicine, Philadelphia, PA 19104, USA; 6 Department of Biomedical Data Sciences, Stanford University School of Medicine, Stanford, CA 94305, USA; 7 Center for Women’s Reproductive Health, Department of Obstetrics and Gynecology, University of Alabama, Birmingham, AL 35294,USA; 8 Department of Medicine, Stanford University School of Medicine, Stanford, CA 94305, USA; * These authors contributed equally to this work. †Present address: Cancer Early Detection Advanced Research Center, Knight Cancer Institute and Department of Molecular and Medical Genetics, Oregon Health and Science University, Portland, OR 97239, USA; ‡ These authors contributed equally to this work

## Abstract

Noninvasive blood tests that provide information about fetal development and gestational age could potentially improve prenatal care. Ultrasound, the current gold standard, is not always affordable in low-resource settings and does not predict spontaneous preterm birth, a leading cause of infant death. In a pilot study of 31 healthy pregnant women, we found that measurement of nine cell-free RNA (cfRNA) transcripts in maternal blood predicted gestational age with comparable accuracy to ultrasound but at substantially lower cost. In a related study of 38 women (25 full-term and 13 preterm deliveries), all at elevated risk of delivering preterm, we identified seven cfRNA transcripts that accurately classified women who delivered preterm up to 2 months in advance of labor. These tests hold promise for prenatal care in both the developed and developing worlds, although they require validation in larger, blinded clinical trials.

Understanding the timing and programming of pregnancy has been a topic of interest for thousands of years. The ancient Greeks had surprisingly detailed knowledge of the different stages of fetal development; they proposed mathematical theories to account for the timing of important landmarks of pregnancy, including delivery of the baby *(**1**–**3**).* Although biologists now have detailed cellular and molecular portraits of both fetal and placental development, this knowledge has not yet translated into molecular tests that reliably predict gestational age for individual pregnancies. Blood levels of human chorionic gonadotropin (HCG) and a-fetoprotein are used to detect conception and fetal complications, respectively; however, neither molecule (either individually or in conjunction) establishes gestational age (4, 5).

Ultrasound imaging and/or the patient’s estimate of her last menstrual period are typically used to estimate gestational age, but the former can be expensive and the latter can be imprecise. Inaccurate dating sometimes leads to unnecessary induction of labor and Cesarean sections, extended postnatal care, and/or increased medical expenses (6–9). Current methods to estimate delivery date generally assume normal development and do not account for premature birth, which affects approximately 15 million neonates every year worldwide (10). In the United States (11), premature birth is the leading cause of neonatal death and complications later in life. Two-thirds of these occur spontaneously, and it would be beneficial to be able to identify which pregnancies are at risk *(**12*, *13**).* Efforts to identify genetic causes and risk factors have had limited success (*11*, *14**–**17*), and clinically, transvaginal sonographic cervical length (CL) and cervicovaginal fetal fibronectin (fFN) measurements have low positive predictive value (*21%* for CL and *17%* for fFN) and specificity (52% for CL) *(**18**).*

In previous work, we showed that longitudinal phenotypic changes in both the mother and the fetus could be monitored by noninvasively measuring cell-free RNA (cfRNA) transcripts from fetal tissues in maternal blood (19). Here, we investigated whether this methodology can be developed into blood tests that establish gestational age and estimate the risk of preterm birth. In our initial study, we recruited 31 pregnant women from Denmark who agreed to donate a blood sample during each week of their pregnancy, resulting in a total of 521 samples (Fig. 1). All women delivered at full term, defined as gestational age at delivery of ≥37 weeks, and their medical records showed no unusual health changes during pregnancy (table S1). Each sample was analyzed by highly multiplexed real-time polymerase chain reaction (PCR) using a panel of genes with expression specific to the placenta or to the immune system, or highly enriched in the fetal liver (table S2).

**Table 1:** **Comparison of gestational age estimates using cfRNA and ultrasound.** Distribution of difference between estimates of gestational age, which assume delivery at 40 weeks gestation, and observed gestational age at delivery listed for four distinct methods, where n indicates the number of women included. Gestational age was estimated using cfRNA measurements from the second (T2), third (T3), or both (T2 and T3) trimesters and ultrasound measurements from the first trimester (T1).

METHOD	Δ[observed – expected delivery date (weeks)] (%)				
	< –2	–1 to –2	±1	+1 to +2	> +2
cfRNA (T2, n = 28)	50	18	32	0	0
cfRNA (T3, n = 31)	0	6	23	29	42
cfRNA (T2 and T3, n = 31)	19	6	45	10	20
Ultrasound (T1, n = 31)	0	26	48	23	3

**Fig. 1:**
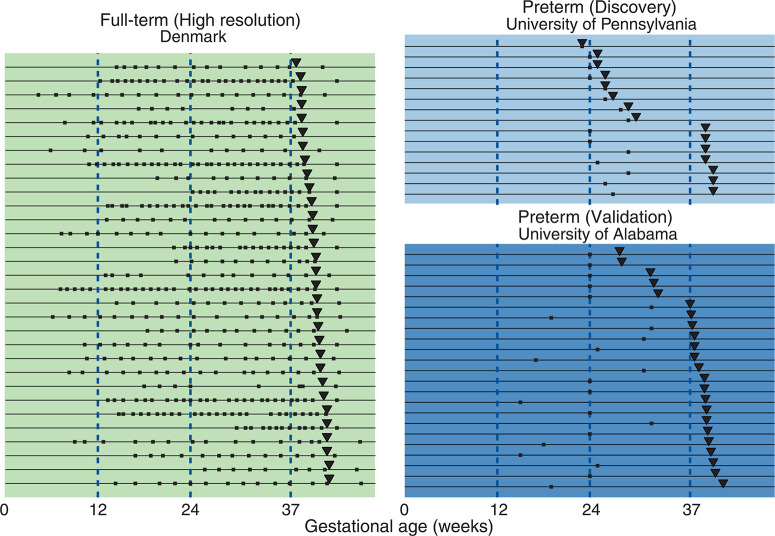
**Sample collection timelines from the Denmark, University of Pennsylvania, and University of Alabama cohorts.** Squares, inverted triangles, and lines indicate sample collection times, delivery dates, and individual women, respectively.

We observed that cfRNA measurements over the course of pregnancy demonstrated differing time courses according to tissue of origin (Fig. 2A and fig. S1). As expected, the levels of cfRNA corresponding to genes specific to the placenta and enriched in the fetal liver increased throughout the course of pregnancy, with the exception of cfRNA corresponding to chorionic gonadotropin b subunit (CGB), which decreased from a peak found in the first trimester. Placental cfRNAs and several fetal liver cfRNAs were not detected above the noise floor after delivery, which supports their pregnancy-derived origin; some fetal liver transcripts were also expressed in the adult liver, and we observed a small maternal baseline for this subset. cfRNA measurements corresponding to immune system–related genes increased during gestation and showed a return to measurable baselines after delivery, which supports their predominantly maternal origin. The body mass index of the mother did not significantly affect cfRNA levels (see supplementary text). Using estimates of cfRNA concentrations in blood across all genes and all pregnancies (Fig. 2B and fig. S2), we discovered that genes within each set (i.e., placental, immune, and fetal) were highly correlated with each other [Median Pearson correlation *r* = 0.79 (placental), 0.79 (immune), 0.74 (fetal), *s* < 10^−14^] and that placental and fetal cfRNA were weakly cross-correlated (*s* = 0.47, *P* < 10^−15^). These findings suggested that cfRNA corresponding to placental genes might provide an accurate estimate of fetal development and gestational age throughout pregnancy.

**Fig. 2:**
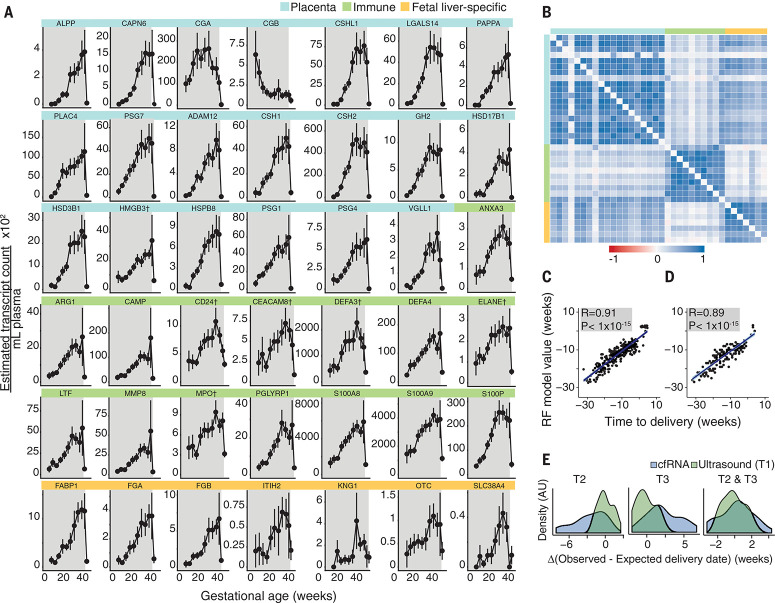
**Application of cfRNA measurements to predict gestational age.** (**A**) For each gene, cfRNA transcript count measurements are shown over the course of gestation. Each point represents the mean cfRNA value ± SEM for either 31 women or 21 women (the latter denoted by †). The antepartum period is highlighted in gray. Placental, immune, and fetal liver–specific genes are highlighted in blue, green, and orange, respectively. (**B**) Heat map of the Pearson correlation coefficient for each gene pair shows that placental, immune, and fetal liver–specific cfRNA [same group colors as (A)] measurements are highly correlated with each other [median Pearson correlation *r* = 0.79 (placenta), 0.79 (immune), 0.74 (fetal liver); *P* < 10^−14^]. Placental and fetal liver–specific genes also show a weak degree of cross-correlation (*r* = 0.47, *P* < 10^−15^). Gene order matches order shown in (A), omitting genes denoted by † in (A). (**C**) Cross-validated random forest (RF) model predicts time to delivery from sampling time point (*R* = 0.91, *P* < 10^−15^, *n* = 21) for training cohort. (**D**) Cross-validated random forest model predicts time to delivery from sampling time point (*R* = 0.89, *P* < 10^−15^, *n* = 10) for validation cohort. (**E**) Distribution of difference in weeks between observed and predicted gestational age at delivery using cfRNA measurements from the second (T2), third (T3), or both (T2 & T3) trimesters (left to right) versus using ultrasound measurements from the first trimester (T1). AU, arbitrary units.

We then built a random forest model to predict time from sample collection until delivery, using cfRNA measurements as the primary features. We trained and validated this model using data from the Danish cohort from 21 women (n=306 blood samples) for training, and from 10 women (n = 215 blood samples) for validation. During training, we applied best-subset selection with 10-fold cross-validation repeated 10 times (see supplementary materials) to identify nine cfRNAs that are specific to the placenta (*CGA, CAPN6, CGB, ALPP, CSHL1, PLAC4, PSG7, PAPPA, and LGALS14)* and together provided equivalent predictive power to the full panel of 51 measured cfRNAs (fig. S3). Our model’s predictions agreed with observed values for both training (Pearson correlation *r* = 0.91, *P* < 10^−15^) (Fig. 2C) and validation sets (*r* = 0.89, *P* < 10^−15^) (Fig. 2D). We also found that model performance improved significantly over the course of pregnancy, as measured by root mean squared error (RMSE) for both training [RMSE = 6.0 (first trimester, T1), 3.9 (second trimester, T2), 3.3 (third trimester, T3), 3.7 (postpartum, PP) weeks] (Fig. 2C) and validation sets [RMSE = 5.4 (T1), 4.2 (T2), 3.8 (T3), 2.6 (PP) weeks] (Fig. 2D). Although distinct subsets of the nine cfRNAs listed above were sufficient to predict time until delivery for subpopulations of women (i.e., nulliparous or multiparous women), we found that all nine genes identified were necessary for accurate prediction across subgroups (see supplementary text).

**Fig. 3:**
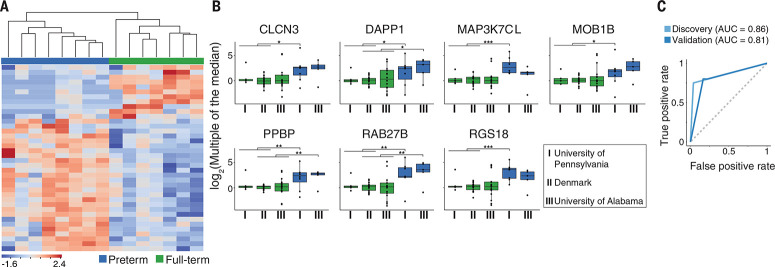
**Application of cfRNA measurements to predict risk of spontaneous preterm delivery.** (**A**) Heat map of the z-scores for 38 differentially expressed genes identified using cfRNA-seq (*P* < 0.001, exact test, likelihood ratio test, and quasi-likelihood F test) shows that genes distinguish women who delivered spontaneously preterm from women who delivered at full term. The two groups of women were separated using hierarchical clustering. (**B**) Means ± SD for differentially expressed genes validated using qRT-PCR in the discovery [University of Pennsylvania (I) and Denmark (II)] and validation [University of Alabama (III)] cohorts. *P < 0.05, **P < 0.01, ***P < 0.0005 (Fisher exact test). (C) Receiver operating characteristic curves for classifier designed to separate women who deliver spontaneously preterm from women who deliver at full term for both the discovery cohort (University of Pennsylvania and Denmark, AUC = 0.86) and the validation cohort (University of Alabama, AUC = 0.81).

The model’s two most important features, *CGA* and *CGB,* encoding chorionic gonadotropin a and β3 subunits of HCG, are known contributors to pregnancy initiation (20) and behaved consistently with what is known from HCG levels during pregnancy (21). Other genes included in the model, such as *PAPPA* (pregnancy-associated plasma protein A), are associated with pregnancy risks such as preterm birth (22).

We next compared our model to other established tools used to predict gestational age (Fig. 2E). In previous studies, ultrasound and last menstrual period estimates of gestational age, which assume delivery at 40 weeks gestation, fell within 14 days of the observed gestational age at delivery with 57.8% and 48.1% accuracy, respectively (7). In this study, for all 31 Danish women, cfRNA estimates of gestational age averaged over a given trimester fell within 14 days of the observed gestational age at delivery with 32% (T2), 23% (T3), and 45% (T2 and T3) accuracy, as compared to 48% (T1) for ultrasound (Table 1). Our results are thus generally comparable to ultrasound measurements, can be performed throughout pregnancy, and do not require a priori physiological knowledge such as the woman’s last menstrual period.

Although the first-generation random forest model predicted time until delivery for full-term pregnancies, we were also interested in testing its performance to predict spontaneous preterm delivery (defined as spontaneous delivery earlier than 37 weeks; see supplementary materials). To explore this question, we studied two separate cohorts, one recruited by the University of Pennsylvania (n = 15) and the other by the University of Alabama at Birmingham (*n* = 23). All of the women in both of these cohorts were already known to be at elevated risk of preterm delivery because they had premature contractions (Pennsylvania) or had a prior spontaneous preterm delivery (Alabama) (Fig. 1, table S1, and supplementary materials). All women in the Alabama cohort and three women in the Pennsylvania cohort received progesterone injections because of a prior spontaneous preterm delivery. All women went into labor spontaneously.

We discovered that although the model validated performance for full-term pregnancies (*n* = 23, RMSE = 4.3 weeks) in these cohorts, it generally failed to predict time until delivery for preterm deliveries (*n* = 13, RMSE = 11.4 weeks) (fig. S4). This suggests that the model’s content may not account for the various outlier physiological events that may lead to preterm birth. This conclusion is supported by the observation that pharmacological agents designed to stop or slow uterine contractions prevent only a small number of preterm deliveries (23, 24).

To identify cfRNA transcripts that might be able to discriminate a spontaneous preterm delivery from a full-term delivery, we performed unblinded RNA sequencing (RNA-seq) on plasmaderived cfRNA collected from women who delivered at full term (n = 7) and preterm (n = 8) in a preterm-enriched cohort (Pennsylvania) (Fig. 1 and table S1). Analysis of RNA-seq data indicated that 38 genes could separate full-term from preterm births with statistical significance (*P* < 0.001; see supplementary materials) (Fig. 3A). We then created a PCR panel to measure the 38 cfRNAs identified by RNA-seq and other immune and placental genes (table S2). We confirmed that the differential expression observed using RNAseq was also observed with quantitative reverse transcription PCR (qRT-PCR) (*r* = 0.72, *P* < 10^−15^) (fig. S5).

We then developed a classifier to identify women who are at risk of preterm delivery and found that using the top seven cfRNAs from the panel (CLCN3, DAPP1, PPBP, MAP3K7CL, MOB1B, RAB27B, and RGS18) [false discovery rate (FDR) ≤ 5%, Hedges’ g ≥ 0.8] (Fig. 3B) in unique combinations of three (table S3 and supplementary materials) accurately classified 6 of 8 preterm samples (75%) and misclassified only 1 of 26 full-term samples (4%) from the Pennsylvania and Denmark cohorts, with a mean area under the curve (AUC) of 0.86 (Fig. 3C). In validation using a preterm-enriched independent cohort (the Alabama cohort), the test accurately classified 4 of 5 preterm samples (80%) and misclassified 3 of 18 full-term samples (17%) (Fig. 1), with a mean AUC of 0.81 (Fig. 3C), using samples collected up to 2 months in advance of labor. Several of the cfRNAs used to predict spontaneous preterm delivery were also individually elevated in women who delivered preterm (FDR ≤ 5%, Hedges’ g ≥ 0.8), demonstrating the robustness of their effect (Fig. 3B). Note that the differences in cfRNA levels cannot be accounted for entirely by progesterone injections, because every woman in the Alabama cohort received injections and the same differences between groups were observed.

Further investigation of the seven genes corresponding to the transcripts identified above revealed that most are ubiquitously expressed, with the exception of a member of the RAS oncogene family (RAB27B), which encodes a protein that regulates placental development (25) and the gene encoding pro-platelet basic protein (*PPBP*). Other protein products encoded by *DAPP1* (dual adaptor of phosphotyrosine 3-phosphoinositides 1) (26), RGS18 (regulator of G protein signaling 18) (27), CLCN3 (chloride voltage-gated channel 3) (28, 29), and *MOB1B* (MOB kinase activator 1B) (30) are indirectly implicated in pregnancy through inflammation (*DAPP1, RGS18*), labor (*CLCN3*), and development (*MOB1B*).

The cfRNA results can be compared to efforts to estimate preterm risk using mass spectroscopic measurements of the ratio of two proteins in blood [SHBG (sex hormone binding globulin) and IBP4 (insulin-like growth factor binding protein 4)] (*31*) or CL and fFN measurements (*18*). In this pilot study, our blood test yielded higher mean accuracy than the mass spectroscopic approach for comparable sample sizes in the validation cohorts [AUC = 0.81 (cfRNA), AUC = 0.67 (IBP4/SHBG)]. When compared to CL and fFN measurements for symptomatic high-risk women experiencing preterm labor, the PCR-based test had a higher positive predictive value [17% (CL), 21% (fFN), 75% (cfRNA, discovery), 80% (cfRNA, validation)] *(**18**).*

Our study has important limitations. Before a diagnostic or screening test based on this work can be used in the clinic, a blinded clinical trial with a larger sample size and diverse ethnicities is essential. Our pilot studies included one Caucasian cohort and two African-American cohorts; data from other ethnic groups would be valuable. Another limitation is that the preterm risk cohorts were all recruited on the basis of elevated risk for preterm birth; it will be important to investigate the performance of the blood test in a broader, unselected population.

Our pilot studies have shown that noninvasive blood tests were able to predict gestational age and identify women at risk of preterm delivery from the same blood sample. These cfRNA PCR-based tests have two advantages over alternatives: broader applicability and lower cost. They can be applied across the globe as a complement to or substitute for ultrasound, which can be expensive and inaccurate during the second and third trimesters (32). Conceivably, similar approaches will prove to be useful for identifying and monitoring fetuses with congenital defects that can be treated in utero—a rapidly growing area of fetal medicine.

## Data and materials availability

cfRNA sequencing data have been deposited in the Sequence Read Archive (SRA) under study accession number SRP130149. qRT-PCR raw and processed data and code used in this manuscript are available at https://github.com/miramou/pregnancy_cfRNA.
