# Supplementary material for: Noninvasive blood tests for fetal development predict gestational age and preterm delivery
Source: Science. 2018 Jun 8;360(6393):1133–6. doi: 10.1126/science.aar3819 (PMC7734383; doi:10.1126/science.aar3819)
Supplement: Supplementary file 1 [file MEDICINE-360-1133-s001.pdf]

www.sciencemag.org/content/360/6393/1133/suppl/DC1

Supplementary Materials for

Noninvasive blood tests for fetal development predict gestational age and preterm delivery

Thuy T. M. Ngo, Mira N. Moufarrej, Marie-Louise H. Rasmussen, Joan Camunas-Soler, Wenying Pan, Jennifer Okamoto, Norma F. Neff, Keli Liu, Ronald J. Wong, Katheryne Downes, Robert Tibshirani, Gary M. Shaw, Line Skotte, David K. Stevenson, Joseph R. Biggio, Michal A. Elovitz, Mads Melbye,* Stephen R. Quake*

*Corresponding author. Email: quake@stanford.edu (S.R.Q.); mmelbye@stanford.edu (M.M.)

Published 8 June 2018, *Science* **360**, 1133 (2018)
DOI: 10.1126/science.aar3819

This PDF file includes:

Materials and Methods

Supplementary Text

Figs. S1 to S5

Tables S1 to S3

References

Materials and Methods

Spontaneous preterm birth definition

Preterm birth is defined as delivery at <37 completed weeks of pregnancy. Spontaneous preterm birth includes preterm labor, preterm spontaneous rupture of membranes, preterm premature rupture of membranes (PPROM) and cervical weakness. It does not include indicated preterm delivery for maternal or fetal conditions.

Study design

This observational study aimed to determine whether cell-free RNA derived from pregnant women could (1) predict time to delivery and (2) distinguish women antepartum who deliver spontaneously preterm from those who deliver at full-term. To do so, we studied three cohorts at hospitals across Denmark, the University of Pennsylvania, and the University of Alabama at Birmingham. The Institutional Review Board approved the study at each collection site, and written consent was obtained for all participants. Only participants above 18 years of age were eligible, and all pregnancies were singleton.

In Denmark, 31 pregnant women receiving routine antepartum care were followed longitudinally until three weeks postpartum. Blood samples were collected weekly antepartum and once three weeks postpartum. All women delivered at full-term (≥ 37 weeks gestation) and were included in subsequent analyses.

At the University of Pennsylvania, pregnant women who came into the clinic with symptoms making them at risk of delivering preterm donated blood once. Of these women, 14 then delivered spontaneously preterm and 14 delivered at full-term. All women entered labor spontaneously. Only women who delivered spontaneously preterm as defined above were included in subsequent analyses. One woman who experienced preeclampsia was excluded from further analyses. Three women had experienced a prior spontaneous preterm birth, and received progesterone injections as per the standard of care.

At the University of Alabama at Birmingham, 26 pregnant women who experienced a prior spontaneous preterm delivery donated blood once. Of these women, eight delivered spontaneous preterm and 18 delivered at full-term. Three women who had blood collected more than nine weeks prior to delivery were excluded from further analyses. All women received progesterone injections as per the standard of care.

Demographics and pregnancy characteristics for all participants included (see Methods below for further technical exclusion criteria) in the final analysis are summarized in table S1.

Sample collection

Blood samples were collected into EDTA-coated Vacutainer tubes and processed within 8 hours of sample collection. Until processing the samples were stored at 4 degrees Celsius. Plasma was separated from blood using standard clinical blood centrifugation protocol.

Gestational age (GA) estimation

The American Congress of Obstetricians and Gynecologists (ACOG) guidelines were used to define accurate gestational age dating for all three cohorts.

cfRNA isolation

cfRNA was extracted from 0.75 to 3 mL of plasma using Plasma/Serum Circulating RNA and Exosomal Purification kit (Norgen, cat 42800). The residue of DNA was digested using Baseline-ZERO DNase (Epicentre) and then cleaned up using RNA Clean and Concentrator-5 kit (Zymo). RNA was eluted in 12 μL elution buffer. 12 of 27 samples from the University of Pennsylvania yielded low cfRNA concentration and were excluded from further analyses.

RT-qPCR assay

RT-qPCR assays consisted of two main reactions: reverse transcription and preamplification of extracted cfRNA and qPCR of pre-amplified cDNA. The primers for our gene panels were designed and synthesized by Fluidigm (table S2). Either 1 to 2 μL or 10 μL out of 12 μL of total purified RNA was used for reverse transcription and preamplification reaction using the CellsDirect One-Step RT-qPCR kit (Thermo-Fisher) and a pool of 96 primer pairs. Preamplification was performed for 20 cycles and residual pooled primers of the reaction were digested using exonuclease I treatment. Multiplex qPCR reactions of 96 samples for 96 primer pairs were performed using the 96x96 dynamic array chip on the Fluidigm Biomark system. Threshold cycles (C_t values) of qPCR reactions were extracted using Fluidigm real-time PCR analysis software.

cfRNA-seq library preparation

cfRNA sequencing libraries were prepared by SMARTer Stranded Total RNAseq - Pico Input Mammalian kit (Clontech) from 6 μL of eluted cfRNA according to the manufacturer's instructions. Short read sequencing were performed on Illumina NextSeq 500 (2x75 bp) platform to a depth of more than 10 million reads per sample.

cfRNA-seq read mapping and quantification

15 cfRNA samples (7 full-term and 8 preterm) collected at the University of Pennsylvania were sequenced. The sequencing reads were mapped to human reference genome (hg38) using STAR aligner. Duplicates were removed by Picard and then unique reads were quantified using htseq-count.

RT-qPCR sample analyses

Raw threshold cycles (C_t values) were quantified in absolute terms. Absolute quantification estimated the transcript counts contained in each sample based on C_t values for known quantities of External RNA Controls Consortium (ERCC) RNA control (fig. S2). Estimated transcript counts were then adjusted for dilution and sample volume and normalized to the total volume of processed plasma. 31 of 31, 23 of 23, and 13 of 15 samples (5 full-term, 8 preterm) collected in Denmark, at the University of Alabama at Birmingham, and at the University of Pennsylvania, respectively, were analyzed using RT-qPCR.

Statistical analysis

Random forest model training

Recursive feature selection (a modification of best subset selection) and model training were performed in parallel in R using the caret package (*33*). Antepartum data from the Danish cohort were smoothed using a three-week centered moving average, and both antepartum and postpartum data were then randomly partitioned into a training set of 21 women and a validation set of 10 women. Models were trained to predict time to delivery, an objective criterion independent of ultrasound-estimated GA, defined as the difference between the GA at sample collection and GA at delivery. Model selection was performed on training data using 10-fold cross-validation repeated 10 times.

Random forest model evaluation

The model was evaluated on a randomly partitioned validation set of 10 women from the Denmark cohort. Model predictive performance was evaluated relative to observed values for any given sample using two metrics: (1) Pearson correlation coefficient and (2) Root mean squared error (RMSE). The Pearson correlation coefficient was calculated on all training or validation data, respectively. RMSE values were calculated by trimester to further characterize model performance over time.

Time to delivery estimation using cfRNA

Recursive feature selection and model training were performed in parallel in R on the same training and validation sets as described above. Data from the Danish cohort used to estimate expected time to delivery were not smoothed prior to model training.

Following model training, time to delivery estimates were obtained for each blood sample. For a specified time period (T2, T3, or both T2 and T3), these estimates were shifted to a reference time point and averaged using the median, yielding an estimate of the expected time to delivery.

Time to delivery estimation using ultrasound

For each woman, the GA at the same reference time point as above was identified. The difference between the aforementioned GA and 40 weeks was then calculated, and taken as the estimate of expected time to delivery using ultrasound.

Comparing cfRNA and ultrasound derived time to delivery estimates

The observed time to delivery for every woman at the same reference time point as above was obtained. Time to delivery estimates as obtained using either cfRNA or ultrasound were then compared to the observed values as follows. The fraction of predicted values, which were over -2, within -2 to -1, within -1 to 1, within 1 to 2, or over 2 weeks, respectively, of the observed value were calculated.

Preterm biomarker candidate discovery using cfRNA-seq

Differentiating genes between full-term (n=7) and preterm (n=8) samples collected at the University of Pennsylvania were identified using three significance tests (exact test, likelihood ratio test, and quasi-likelihood F-test) in the edgeR package in R (*34–37*). Genes were classified as differentially expressed between women who delivered preterm and full-term if $P<0.001$ for all three aforementioned tests. Counts for differentiating genes were then converted to counts per million (CPM) for each sample, and z-scores were calculated for each gene. Average hierarchical clustering with a Euclidean distance metric was performed to cluster both genes and samples using the seaborn package in Python (*38*).

Preterm biomarker candidate discovery using RT-qPCR

Absolute RT-qPCR values for genes identified using cfRNA-seq as described above were normalized using a modified multiple of the median approach as applied in (*39*). For each cohort, the median for full-term pregnancies was quantified by trimester for each gene. cfRNA transcript counts were then divided by the median calculated above to obtain multiple of the median values.

Data for each gene were then separated based on a cutoff value of 2.5 to create a two-dimensional contingency table, and a one-tailed Fisher exact test was performed to compare women who delivered full-term and preterm in the University of Pennsylvania cohort. An effect size as calculated by Hedges' g was also obtained for every gene. Genes were considered significantly different between women who delivered preterm and full-term using an effect size threshold of 0.8 and a false discovery rate (FDR) of 5% as described previously (*40*).

Candidate biomarkers were then combined into unique groups of three. For each woman, a combination classified her as at risk for preterm delivery if the multiple of the median for all three cfRNAs was greater than 2.5. Combinations with a true positive rate of greater than 0.75 and a false positive rate less than 0.05 were selected as predictive of spontaneous preterm birth.

Preterm cfRNA gene-transcript candidate validation using RT-qPCR

Using the combinations of candidate biomarkers identified during discovery, each woman was then assigned a probability of delivery preterm based on the fraction of combinations, which classified her as at risk for preterm delivery. Each combination classified a woman as at risk for preterm delivery using the same metric as described above.

Data availability and reproducibility

RT q-PCR raw and processed data as well as code used in this manuscript are available at https://github.com/miramou/pregnancy_cfRNA. cfRNA sequencing data have been deposited in the Sequence Read Archive (SRA) under study accession number SRP130149.

Supplementary Text

Supplementary note 1: Body mass index (BMI) does not affect cell-free RNA (cfRNA) levels

We have tested for the effect of BMI on circulating cfRNA levels using estimated transcript counts of GAPDH per milliliter of plasma and found no significant difference between underweight (BMI < 18.5), normal weight (18.5 ≤ BMI < 25), overweight (25 ≤ BMI < 30), and obese (BMI ≥ 30) individuals both before and after Bonferroni correction using a Wilcoxon rank sum test.

P-values for distinct tests of GAPDH levels before and after Bonferroni correction, respectively, were as follows: (1) underweight versus normal weight (P = 0.58, 1), underweight versus overweight (P = 0.12, 0.80), underweight versus obese (P = 0.26, 1), normal weight versus overweight (P = 0.06, 0.35), normal weight versus obese (P = 0.16, 0.95), and overweight versus obese (P = 0.72, 1). Similar results were obtained for placental-specific cfRNAs such as CAPN6, CGA, and CGB.

All comparisons were done within cohorts so that differences in BMI distribution between cohorts were not confounding.

Supplementary note 2: Transcriptomic signature differences between subpopulations when predicting time to delivery

We trained several distinct models on subpopulations of women (i.e., nulliparous or multiparous women, women carrying male or female fetuses) to determine the importance of the 9 genes that compose the transcriptomic signature identified. Training 4 distinct models for women carrying male or female fetuses and nulliparous or multiparous women revealed that 2 of the 9 genes identified in the main text were sufficient to predict time to delivery for women carrying male (CGA, CSHL1) or female (CGA, CAPN6) fetuses and multiparous (CGA, CSHL1) women. However, all 9 genes were necessary to predict time until delivery for nulliparous women, highlighting the importance of the transcriptomic signature identified.

Fig. S1

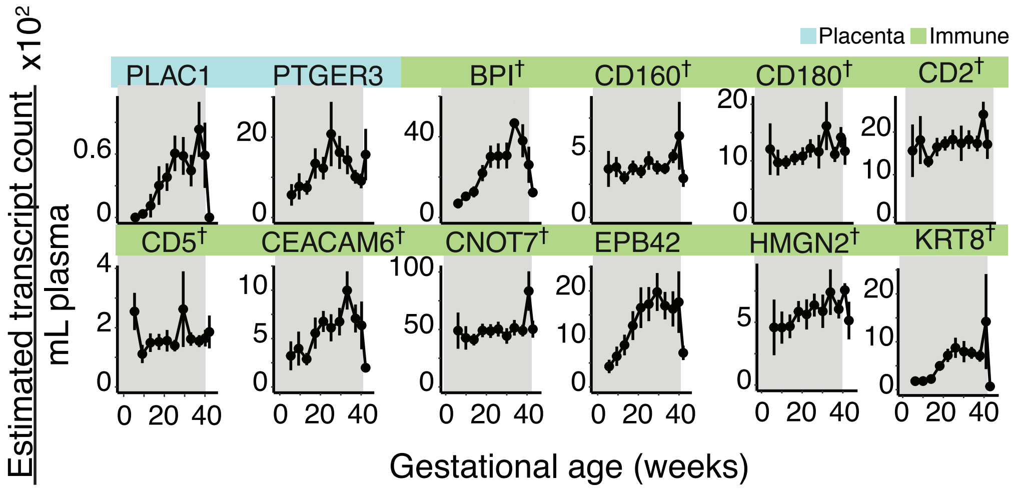

Plots of additional cfRNA transcript dynamics over the course of pregnancy. For each gene, cfRNA transcript count measurements are shown over the course of gestation. Each point represents the mean cfRNA value ± SEM for either 31 women or 21 women (denoted by †). The antepartum period is highlight in gray. Placental and immune genes are highlighted in blue and green, respectively.

Fig. S2

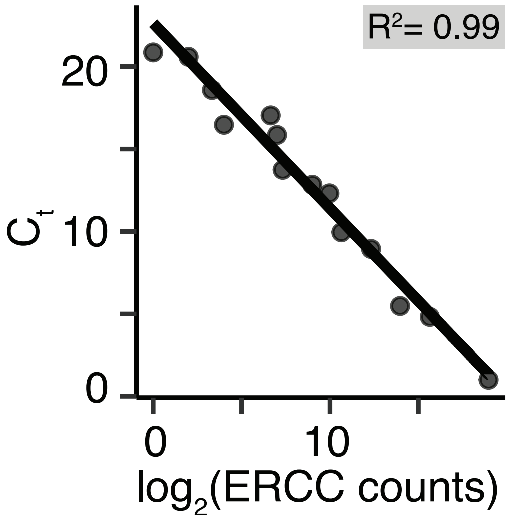

Linear regression for threshold cycle (C_t) versus External RNA Controls Consortium (ERCC) RNA control shows that measured values agree with linear fit (R-squared=0.99).

Fig. S3

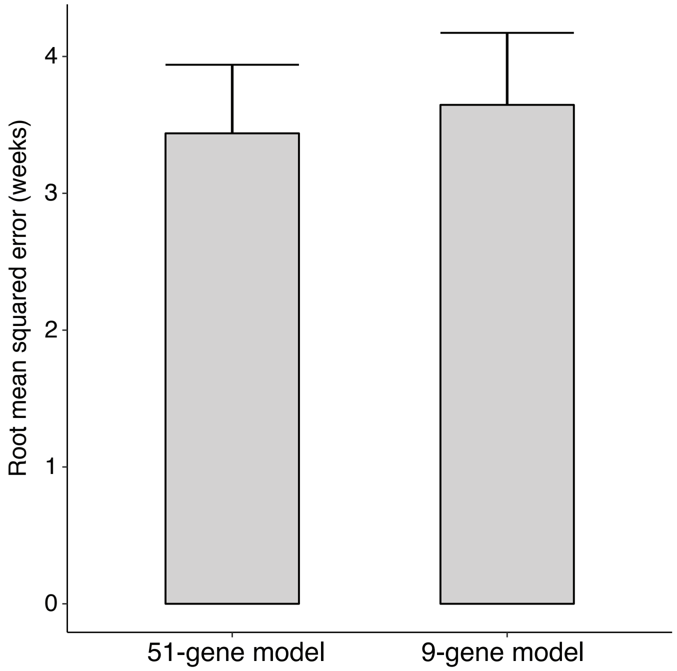

Random forest model trained on a 9-gene subset performs comparably to model trained using full gene set.

Fig. S4

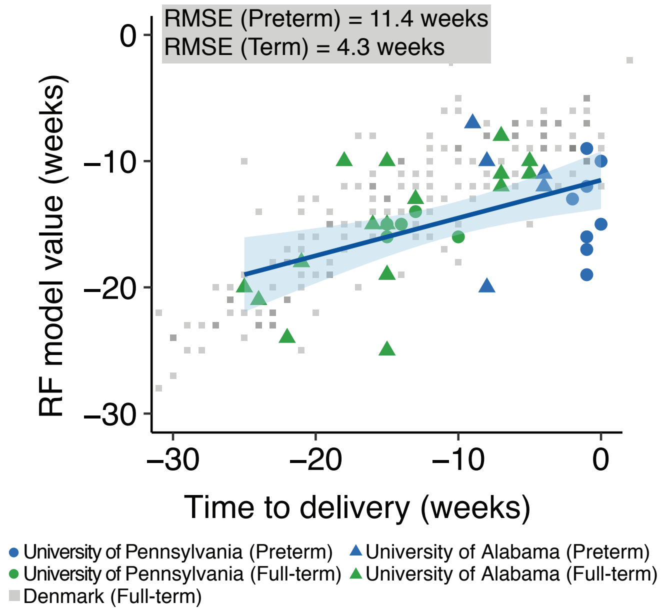

Cross-validated random forest model successfully predicts time to delivery from sampling time point for women who deliver full-term (RMSE = 4.3 weeks, n = 23), but fails to predict time to delivery for women who deliver preterm (RMSE = 11.4, n = 13) in two independent test cohorts (University of Pennsylvania and University of Alabama).

Fig. S5

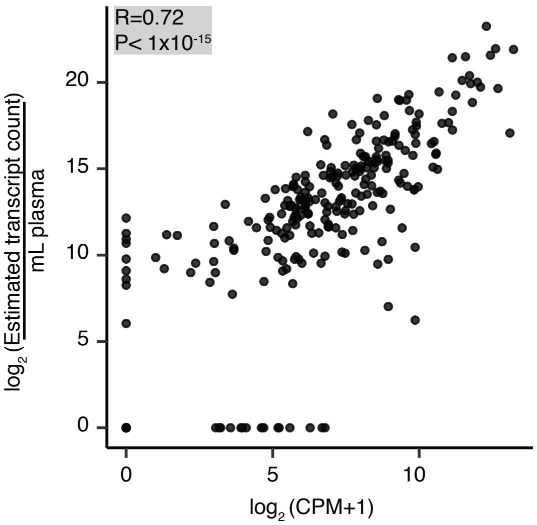

cfRNA measurements for genes identified as differentially expressed using cfRNA-seq agree with measurements using RT-qPCR (Pearson coefficient r = 0.72, $P < 1x10^{-15}$).

Table S1.

Participant and pregnancy characteristics.

**Demographics**	**Denmark (n=31)**	**Pennsylvania (n = 15)**		**Alabama (n = 23)**	
	Full-term	*Preterm*	*Full-term*	*Preterm*	*Full-term*
	(n = 31)	*(n = 8)*	*(n = 7)*	*(n = 5)*	*(n = 18)*
Age (years, mean±SD)	29.9±3.2	23.0±5.7	23.7±3.4	25.2±2.8	25.8±4.4
Parity (% nulliparous)	19 (61.3)	4 (50.0)	3 (42.9)	0 (0)	0 (0)
BMI (kg/m^2, mean±SD)	22.1±3.6	25.1±5.0	31.9±5.7	33.0±11.3	28.6±7.0
Ethnicity (% Hispanic)	0 (0)	0 (0)	0 (0)	0 (0)	0 (0)
Caucasian (%)	31 (100)	0 (0)	0 (0)	0 (0)	1 (6)
African-American (%)	0 (0)	8 (100)	7 (100)	5 (100)	17 (94)
Gestational age at delivery (weeks, mean±SD)	40±1.2	26.4±2.3	39.4±0.5	30.6±2.4	38.7±1.2
Mode of delivery					
Vaginal	23 (74.2)	5 (62.5)	7 (100)	5 (100)	16 (29)
Cesarean-section	8 (25.8)	3 (37.5)	0 (0)	0 (0)	2 (11)
Gender (% male)	14 (45.2)	4 (50)	4 (57.1)	3 (60)	10 (56)
Birthweight (kg, mean±SD)	3.6±0.6	0.9±0.3	3.4±0.4	1.7±0.7	3.1±0.4

Table S2.

Gene primer sequences used in RT-qPCR.

Gene	Forward Primer	Reverse Primer
ACTB	CCAACCGCGAGAAGATGAC	TAGCACAGCCTGGATAGCAA
ADAM12	TGAGAAAGGAGGCTGCATCA	CTGCTGCAACTGCTGAACA
AFP	GCCTCTTCCAGAAACTAGGAGAA	GGGGCTTTCTTTGTGTAAGCAA
ALPP	GACAGCTGCCAGGATCCTAA	GTCTGGCACATGTTTGTCTACA
ANXA1	AAGTGCGCCACAAGCAAA	TGCCTTATGGCGAGTTCCA
ANXA3	CAGCGGCAGCTGATTGTTAA	CAGAGAGATCACCCTTCAAGTCA
APLF	ACCCAGATGACTCCCACAAA	CAAGGATTGGCTGCTGCTTA
APOA4	AAGGCCGTGGTCCTGAC	TCAGCTGGCTGAAGTAGTCC
ARG1	GCAAGGTGGCAGAAGTCAA	ATGGCCAGAGATGCTTCCA
AVPR1A	GCGCCTTTCTTCATCATCCA	GATGGTGATGGTAGGGTTTTCC
BPI	TCCTGGAACTGAAGCACTCA	GCAGCACAAGAATGGGTACA
CALCB	CCCCTTCCTGGCTCTCAGTA	GGTCTGGGCTGCTCTCCA
CAMP	GGACAGTGACCCTCAACCA	CAGCAGGGCAAATCTCTTGTTA
CAPN6	TGGAAAGGTGGTGTGGAAAC	GTCAGCTGGTGGTTGCTAA
CCL20	TGATGTCAGTGCTGCTACTCC	CTGTGTATCCAAGACAGCAGTCA
CD160	CTCAGTTCAGGCTTCCTACA	TCTTTTGGCACAAGGCTTAC
CD180	CACAATAGAACCTTCAGCAGAC	GAAAAGTGTCTTCATGTATCCAGTTA
CD2	ATTCCAGCTTCAACCCCTCA	ATGACTAGGTGCCTGGGAAC
CD24	CCAACTAATGCCACCACCAA	CGAAGAGACTGGCTGTTGAC
CD5	CCCCTTGCCTACAAGAAGCTA	TCCCGTTGGGCCAATCC
CDK5R1	AGCAAGAACGCCAAGGA	CGGCCACGATTCTCTTCCAA

	CAA	
CEACAM6	AGATTGCATGTCCCCTGGAA	GGGTGGGTTCCAGAAGGTTA
CEACAM8	TATGCCTGCCACACCACTAA	GCCAGGAGAACTTCCTTGTACTA
CGA	TCAACCGCCCTGAACACA	ACACCGACAATGTGACCAGAA
CGB	AGCCTTCCAAGCCCATCC	TGCGGATTGAGAAGCCTTTA
CLCN3	CGTGGTCAGGATGGCTAGTA	CCAATCGGCAGCAATGTCTA
CNOT7	GTCCTCTGTGAAGGGGTCAAA	TCTTCAGGCAAGTTAGAGTTGGTTA
COL17A1	TGACAACCCAGAGCTCATCC	GGACGCCATGTTGTTTGGAA
COL21A1	CGTCCAGGTGTCAGAGGATTA	ACCTTGTTCTCCAGGATACCC
CPVL	TGAAGTGGCTGGTTACATCC	AGAGGCTGGTCATAGGGTAA
CRP	GTCTTGACCAGCCTCTCTCA	ACGGTGCTTTGAGGGATACA
CSH1	ACAAGAGACCGGCTCTAGGA	TTGCCACTAGGTGAGCTGTC
CSH2	CGTTCCGTTATCCAGGCTTTT	ACTCCTGGTAGGTGTCAATGG
CSHL1	TTAGAGCTGCTCCACATCTCC	ACCAGGTTGTTGGTGAAGGTA
CUX2	TCCATCACCAAGAGGGTGAA	CAGGATGCTTTCCCCAAACA
CYP3A7	ACGTGCATTGTGCTCTCTCA	CAGCACTGATTTGGTCATCTCC
DAPP1	TGGGCACCAAAGAAGGTTA	TTCCTGTGCAGAGTAAACCA
DCX	ATCTCTACGCCCACCAGTCC	AGCGAGTCCGAGTCATCCAA
DEFA3	GACGAAAGCTTGGCTCCAAA	GTTCCATAGCGACGTTCTCC
DEFA4	TGGGATAAAAGCTCTGCTCTTCA	TGTTCGCCGGCAGAATACTA
DGCR14	ACAAGGCCAAGAATTCCCTCA	TGCCGGGGCTTCTTAAACA
DLX2	TTCGTCCCCAGCCAACAA	TGGCTTCCCGTTCACTATCC
EGFR	GCAGTGACTTTCTCAGCAACA	TTGGGACAGCTTGGATCACA
ELANE	CTCTGCCGTCGCAGCAA	TGGATTAGCCCGTTGCAGAC
ENAH	GCCGGAGCAAAACTTAGG	AGGCGGAGTTCACACCAATA

	AAA	
EPB42	GCCAAGCTCTGGAGGAAGAA	GAGAAGAACAGGCCGATGGTTA
EPOR	ATCCTGGTGCTGCTGAC	GGCCAGATCTTCTGCTTCA
EPX	AGTTCAGAAGAGCCCGAGAC	GCGCTGTCTTTTGGTGAAAAC
EVX1	TACCGGGAGAACTACGTATCCA	ATGCGCCGGTTCTGGAA
FABP1	AGGAATGTGAGCTGGAGACA	TTGTCACCTTCCAACTGAACC
FABP7	GCTACCTGGAAGCTGACCAA	CCACCTGCCTAGTGGCAAA
FAM212B-AS1	GGAAAGGGGTGGATGTGTCA	CACCCAGGATGTCCTTGTTCTA
FGA	ATGTTAGAGCTCAGTTGGTTGATA	TACTGCATGACCCTCGACAA
FGB	ATATTGTCGCACCCCATGCA	ACCTCCTTTCCTGATAATTTCCTCAC
FOXG1	GCCAGCAGCACTTTGAGTTA	TGAGTCAACACGGAGCTGTA
FRMD4B	GAAACCCAGCCAGAAAGCAA	AGGTGGTGGTGTCAGACAAA
FRZB	CCTCTGCCCTCCACTTAATGTTA	CAGCTATAGAGCCTTCCACCAA
FSTL3	CCGGACCTGAGCGTCATGTA	GCACACCACGTGCTCACA
GAPDH	GAACGGGAAGCTTGTCATCAA	ATCGCCCCACTTGATTTTGG
GCA	TCAGTTTGGAAACCTGCAGAA	GCTGCCCATAGCTCTTTGAA
GH2	CCCGTCGCCTGTACCA	TGTTGGAATAGACTCTGAGAAGCA
GNAZ	CGGCTACGACCTGAAACTCTA	TGAGTGAGGTGTTGATGAACCA
GPR116	CCAGAGGCAGTGCAAACATAA	AGAAATTGGGTCCGGGGTTA
GRHL2	ACTCCGGACAGCACATACA	CCAACTGAAGCACTCCGAAA
GSN	AAGACCTGGCAACGGATGAC	TTGAGAATCCTTTCCAACCCAGAC
GYPB	ACAACTTGTCCATCGTTTCAC	ACCAGCCATCACACACAA
HAL	AGAACTGAACAGCGCAACA	GCTGGGTATTCACCATGGAA
HBG2	GGTGACCGTTTTGGCAAT	CACTGGCCACTCCAGTCAC

	CC	
HIST1H2BM	GCCTGGCGCATTACAACA A	CAATTCCCCGGGTAGCAGTA
HMGB3	CGGCAAAGCTGAAGGAG AAGTA	CAGGACCCTTTGCACCATCA
HMGN2	ACACAGTGCTAGGTGCAG TTA	TCCATACTCCCAGCCTTTCAC
HS6ST1	AAGTTCATCCGGCCCTTC A	GGTGTCTTCATCCACCTCCA
HSD17B1	TGGACGTAAGGGACTCAA AATCC	CCCAGGCCTGCGTTACA
HSD3B1	TGTGCCTTACGACCCATG TA	GTTGTTCAGGGCCTCGTTTA
HSPB8	GCAAGAAGGTGGCATTGT TTCTA	TCTGGGGAAAGTGAGGCAAA
ITIH2	AGAGAAGAGAAGGCTGG TGAAC	TCCAGGTTGTCAGGAGCAAA
KLF9	TCCCATCTCAAAGCCCAT TACA	CTCGTCTGAGCGGGAGAA
KNG1	CTGGCAGGACTGTGAGTA CAA	ATTTCGTACTGCTCCTCTTCCC
KRT8	TGACCGACGAGATCAACT TCC	TGTGCCTTGACCTCAGCAA
KRT81	TGAAGGCATTGGGGCTGT G	AGCCTGACACGCAGAGGT
LGALS14	TGTGCATCTATGTGCGTC AC	GGAATCGATGGGCAAAGTTG TA
LHX2	CAAAAGACGGGCCTCACC AA	CGTAAGAGGTTGCGCCTGAA
LIPC	CATCGGTGGAACGCACAA	GGGCACTTCCCTCAAACAAA
LRRN3	GCCTTGGTTGGACTGGAA AA	TTTGAAGAGCAACATGGGGT AC
LTF	CTCCCAGGAACCGTACTT CA	CTCTGATAAAAGCCACGTCTC C
LYPLAL1	CATCAAGATGTGGCAGGA GTA	TGCAGTACCATGACACTGAAA TA
MAP3K7CL	GACTCCATTCCTTTGGTCT TTCC	CCATGGATTCCTCGGAGTCA
MEF2C	TGGTCTGATGGGTGGAGA CC	TGAGTTTCGGGGATTGCCATA C
MMD	TCTCACAATGGGATTCTC TCCA	CAGGCAAGTTCCTGAAGTCC
MMP8	TGCCGAAGAAACATGGAC CAA	AGCCCCAAAGAATGGCCAAA
MN1	AGAAGGCCAAACCCCAG	ATGCTGAGGCCTTGTTTGC

	AA	
MOB1B	GAGAGTTGTCCAGTGATG TCA	GTCCTGAACCCAAGTCATCA
MPO	CATCGGTACCCAGTTCAG GAA	TGCTGCATGCTGAACACAC
NFATC1	TCCTCTCCAACACCAAAG TCC	AGGATTCCGGCACAGTCAA
NFATC2	TGGAAGCCACGGTGGATA A	TGTGCGGATATGCTTGTTCC
NPY1R	TCTGCTCCCTTCCATTCCC	GAATTCTTCATTCCCTTGAAC TGAAC
NTSR1	CGCCTCATGTTCTGCTAC A	TAGAAGAGTGCGTTGGTCAC
OAZ1	CGAGCCGACCATGTCTTC A	AAGCTGAAGGTTCGGAGCAA
OTC	CCAGGCTTTCCAAGGTTA CCA	TGGCTTTCTGGGCAAGCA
P2RY12	ACTGGATACATTCAAACC CTCCA	TGGTGCACAGACTGGTGTTA
PAPPA	GTACTGTGGCGATGGCAT TATAC	AGAAAAGGGAGCAGCCATCA
PAPPA2	ACAGTGGAAGCCTGGGTT AA	ACAGTGTGGGAGCAGTTATCA
PCDH11X	CTGGCATCCAGTTGACGA AA	CATCAGGGCCTAGCAGGTAA
PGLYRP1	GTGCAGCACTACCACATG AA	TATACGAGCCCGTCTTCTCC
PKHD1L1	GCCAGCTGCTATATCACA CAAA	AAACCCAGGGCTACTTCCAA
PLAC1	GCCACATTTCAAAGGAAA CTGAC	TCCCTGCAGCCAATCAGATA
PLAC4	CCACCAAGAAGCCACTTT CC	TACCAGCAATGCCAGGGTTA
POLE2	AGAAACTGCGTCCGTTTT CC	GGAGTCAGATGTCCTTGGGAT AA
POU3F2	CGGATCAAACTGGGATTT ACCC	CGAGAACACGTTGCCATACA
PPBP	TCTGGCTTCCTCCACCAA A	CAGCGGAGTTCAGCATACAA
PRDX5	GTTCGGCTCCTGGCTGAT	CAAAGATGGACACCAGCGAA TC
PRG2	GGGGCAGTTTCTGCTCTT CA	TCATCCTCAGGCAGCGTCTTA
PSG1	GCAGGATCCTACACCTTA CACA	TGCTGGAGATGGAGGGCTTA

PSG2	CTGGCGAGGAAAGCTCCA	CAGAAATGACATCACAGCTG CTA
PSG4	CTCCCCAGCATTTACCCTT CA	GGTTAGACTCGGCGAAGCA
PSG7	ACCCAGTCACCCTGAATG TC	GCAGGACAAGTAGAGGTTTT GTC
PTGER3	GTCGGTCTGCTGGTCTCC	TGTGTCTTGCAGTGCTCAAC
RAB11A	AGGCACAGATATGGGAC ACA	ATAAGGCACCTACAGCTCCA
RAB27B	ACCAGATCAGAGGGAAG TCA	CAGTTGCTGCACTTGTTTCA
RAP1GAP	GGAAGCAGGATGGATGA ACA	CTCGGGTATGGAATGTAGTCC
RGS18	TGAAGACACCCGCTCCAG TA	CCCCATTTCACTGCCTCTTCA
RHCE	TGGGAAGGTGGTCATCAC AC	CAGCACCCGCTGAGATCA
RNASE2	GCCAAGATCCCATCTCTC CA	AGGCACTTCAGCTCAGGAAA
RPL23AP7	CTGGCTGTGGGTGTGGTA CT	CGCTCCACTCCCTCTAGGC
S100A8	GCTAGAGACCGAGTGTCC TCA	CCAGAATGAGGAACTCCTGG AA
S100A9	TCAAAGAGCTGGTGCGAA AA	ATTTGTGTCCAGGTCCTCCA
S100P	GAAGGAGCTACCAGGCTT CC	AGCAATTTATCCACGGCATCC
SAMD9	CTTCGAGAAGTCTTGCAA CC	GCCAGAATAAGAGGGAAGCT A
SATB2	TTTGCCAAAGTGGCTGCA AA	TTTCTGGGCTTGGGTTCTCC
SEMA3B	TGCACCAGTGGGTGTCAT A	GTGGAACTGAAGGTGCCAAA
SERPINA7	AGAAGTGGAACCGCTTAC TACA	AGTGTGGCTCCAAGGTCATA
SLC12A8	GCTGCCATCGTGTATTTCT ACA	AGACCTCATCCACCGGAAAA
SLC2A2	GGGAGCACTTGGCACTTT TCA	GCAGGATGTGCCACAGATCA
SLC38A4	GGTCCTTCCCATCTACAG TGAA	AGCATCCCCGTGATGGAAATA
SLC4A1	TGCTGCCGCTCATCTTCA	CAAAGGTTGCCTTGGCATCA
SLITRK3	GACCTGGCGCTCCAGTTT A	CCTCTGTGAAGCATCTCAGCT A
TBC1D15	AAGACGGCTTGATTTCAG	GCATCATCCAATGGTCTCCA

	GAA	
TFIP11	TGTTAAGCAGGACGACTT TCC	CCTTTCTGGCTGGGCTTAAA
VCAN	GGTGCCTCTGCCTTCCAA	TTGTGCCAGCCATAGTCACA
VGLL1	AGAGTGAAGGTGTGATGC TGAA	GCACGGTTTGTGACAGGTAC

Table S3.

All combinations of seven cfRNA transcripts used to classify risk of spontaneous preterm delivery in Fig. 3C.

Combination	Gene 1	Gene 2	Gene 3
1	RGS18	DAPP1	PPBP
2	RGS18	RAB27B	PPBP
3	RGS18	MOB1B	PPBP
4	RGS18	PPBP	MAP3K7CL
5	RGS18	PPBP	CLCN3
6	DAPP1	RAB27B	PPBP
7	DAPP1	MOB1B	PPBP
8	DAPP1	PPBP	CLCN3
9	RAB27B	MOB1B	PPBP
10	RAB27B	PPBP	MAP3K7CL
11	RAB27B	PPBP	CLCN3
12	MOB1B	PPBP	MAP3K7CL
13	MOB1B	PPBP	CLCN3

References

1. A. E. Hanson, Paidopoiïa: Metaphors for conception, abortion, and gestation in the Hippocratic Corpus. *Clio Med.* **27**, 291–307 (1995). Medline

2. A. E. Hanson, The eight months' child and the etiquette of birth: Obsit omen! *Bull. Hist. Med.* **61**, 589–602 (1987). Medline

3. H. N. Parker, Greek embryological calendars and a fragment from the lost work of Damastes, "On the care of pregnant women and of infants". *Class. Q.* **49**, 515–534 (1999). doi:10.1093/cq/49.2.515 Medline

4. L. Dugoff, J. C. Hobbins, F. D. Malone, J. Vidaver, L. Sullivan, J. A. Canick, G. M. Lambert-Messerlian, T. F. Porter, D. A. Luthy, C. H. Comstock, G. Saade, K. Eddleman, I. R. Merkatz, S. D. Craigo, I. E. Timor-Tritsch, S. R. Carr, H. M. Wolfe, M. E. D'Alton, Quad screen as a predictor of adverse pregnancy outcome. *Obstet. Gynecol.* **106**, 260–267 (2005). doi:10.1097/01.AOG.0000172419.37410.eb Medline

5. E. Yefet, O. Kuzmin, N. Schwartz, F. Basson, Z. Nachum, Predictive Value of Second-Trimester Biomarkers and Maternal Features for Adverse Pregnancy Outcomes. *Fetal Diagn. Ther.* **42**, 285–293 (2017). doi:10.1159/000458409 Medline

6. W. B. Barr, C. C. Pecci, Last menstrual period versus ultrasound for pregnancy dating. *Int. J. Gynaecol. Obstet.* **87**, 38–39 (2004). doi:10.1016/j.ijgo.2004.06.008 Medline

7. D. A. Savitz, J. W. Terry Jr., N. Dole, J. M. Thorp Jr., A. M. Siega-Riz, A. H. Herring, Comparison of pregnancy dating by last menstrual period, ultrasound scanning, and their combination. *Am. J. Obstet. Gynecol.* **187**, 1660–1666 (2002). doi:10.1067/mob.2002.127601 Medline

8. K. A. Bennett, J. M. G. Crane, P. O'shea, J. Lacelle, D. Hutchens, J. A. Copel, First trimester ultrasound screening is effective in reducing postterm labor induction rates: A randomized controlled trial. *Am. J. Obstet. Gynecol.* **190**, 1077–1081 (2004). doi:10.1016/j.ajog.2003.09.065 Medline

9. M. Whitworth, L. Bricker, C. Mullan, Ultrasound for fetal assessment in early pregnancy. *Cochrane Database Syst. Rev.* **2015**, CD007058 (2015). doi:10.1002/14651858.CD007058.pub3 Medline

10. H. Blencowe, S. Cousens, D. Chou, M. Oestergaard, L. Say, A.-B. Moller, M. Kinney, J. Lawn, Born Too Soon Preterm Birth Action Group, Born too soon: The global epidemiology of 15 million preterm births. *Reprod. Health* **10** (suppl. 1), S2 (2013). doi:10.1186/1742-4755-10-S1-S2 Medline

11. Institute of Medicine (US) Committee on Understanding Premature Birth and Assuring Healthy Outcomes, *Preterm Birth: Causes, Consequences, and Prevention* (National Academies Press, 2007).

12. C. J. Murray, T. Vos, R. Lozano, M. Naghavi, A. D. Flaxman, C. Michaud, M. Ezzati, K. Shibuya, J. A. Salomon, S. Abdalla, V. Aboyans, J. Abraham, I. Ackerman, R. Aggarwal, S. Y. Ahn, M. K. Ali, M. Alvarado, H. R. Anderson, L. M. Anderson, K. G. Andrews, C. Atkinson, L. M. Baddour, A. N. Bahalim, S. Barker-Collo, L. H. Barrero, D. H. Bartels, M. G. Basáñez, A. Baxter, M. L. Bell, E. J. Benjamin, D. Bennett, E. Bernabé, K. Bhalla, B. Bhandari, B. Bikbov, A. Bin Abdulhak, G. Birbeck, J. A. Black, H. Blencowe, J. D. Blore, F. Blyth, I. Bolliger, A. Bonaventure, S. Boufous, R. Bourne, M. Boussinesq, T. Braithwaite, C. Brayne, L. Bridgett, S. Brooker, P. Brooks, T. S. Brugha, C. Bryan-Hancock, C. Bucello, R. Buchbinder, G. Buckle, C. M. Budke, M. Burch, P. Burney, R. Burstein, B. Calabria, B. Campbell, C. E. Canter, H. Carabin, J. Carapetis, L. Carmona, C. Cella, F. Charlson, H. Chen, A. T. Cheng, D. Chou, S. S. Chugh, L. E. Coffeng, S. D. Colan, S. Colquhoun, K. E. Colson, J. Condon, M. D. Connor, L. T. Cooper, M. Corriere, M. Cortinovis, K. C. de Vaccaro, W. Couser, B. C. Cowie, M. H. Criqui, M. Cross, K. C. Dabhadkar, M. Dahiya, N. Dahodwala, J. Damsere-Derry, G. Danaei, A. Davis, D. De Leo, L. Degenhardt, R. Dellavalle, A. Delossantos, J. Denenberg, S. Derrett, D. C. Des Jarlais, S. D. Dharmaratne, M. Dherani, C. Diaz-Torne, H. Dolk, E. R. Dorsey, T. Driscoll, H. Duber, B. Ebel, K. Edmond, A. Elbaz, S. E. Ali, H. Erskine, P. J. Erwin, P. Espindola, S. E. Ewoigbokhan, F. Farzadfar, V. Feigin, D. T. Felson, A. Ferrari, C. P. Ferri, E. M. Fèvre, M. M. Finucane, S. Flaxman, L. Flood, K. Foreman, M. H. Forouzanfar, F. G. Fowkes, M. Fransen, M. K. Freeman, B. J. Gabbe, S. E. Gabriel, E. Gakidou, H. A. Ganatra, B. Garcia, F. Gaspari, R. F. Gillum, G. Gmel, D. Gonzalez-Medina, R. Gosselin, R. Grainger, B. Grant, J. Groeger, F. Guillemin, D. Gunnell, R. Gupta, J. Haagsma, H. Hagan, Y. A. Halasa, W. Hall, D. Haring, J. M. Haro, J. E. Harrison, R. Havmoeller, R. J. Hay, H. Higashi, C. Hill, B. Hoen, H. Hoffman, P. J. Hotez, D. Hoy, J. J. Huang, S. E. Ibeanusi, K. H. Jacobsen, S. L. James, D. Jarvis, R. Jasrasaria, S. Jayaraman, N. Johns, J. B. Jonas, G. Karthikeyan, N. Kassebaum, N. Kawakami, A. Keren, J. P. Khoo, C. H. King, L. M. Knowlton, O. Kobusingye, A. Koranteng, R. Krishnamurthi, F. Laden, R. Lalloo, L. L. Laslett, T. Lathlean, J. L. Leasher, Y. Y. Lee, J. Leigh, D. Levinson, S. S. Lim, E. Limb, J. K. Lin, M. Lipnick, S. E. Lipshultz, W. Liu, M. Loane, S. L. Ohno, R. Lyons, J. Mabweijano, M. F. MacIntyre, R. Malekzadeh, L. Mallinger, S. Manivannan, W. Marcenes, L. March, D. J. Margolis, G. B. Marks, R. Marks, A. Matsumori, R. Matzopoulos, B. M. Mayosi, J. H. McAnulty, M. M. McDermott, N. McGill, J. McGrath, M. E. Medina-Mora, M. Meltzer, G. A. Mensah, T. R. Merriman, A. C. Meyer, V. Miglioli, M. Miller, T. R. Miller, P. B. Mitchell, C. Mock, A. O. Mocumbi, T. E. Moffitt, A. A. Mokdad, L. Monasta, M. Montico, M. Moradi-Lakeh, A. Moran, L. Morawska, R. Mori, M. E. Murdoch, M. K. Mwaniki, K. Naidoo, M. N. Nair, L. Naldi, K. M. Narayan, P. K. Nelson, R. G. Nelson, M. C. Nevitt, C. R. Newton, S. Nolte, P. Norman, R. Norman, M. O'Donnell, S. O'Hanlon, C. Olives, S. B. Omer, K. Ortblad, R. Osborne, D. Ozgediz, A. Page, B. Pahari, J. D. Pandian, A. P. Rivero, S. B. Patten, N. Pearce, R. P. Padilla, F. Perez-Ruiz, N. Perico, K. Pesudovs, D.

Phillips, M. R. Phillips, K. Pierce, S. Pion, G. V. Polanczyk, S. Polinder, C. A. Pope 3rd, S. Popova, E. Porrini, F. Pourmalek, M. Prince, R. L. Pullan, K. D. Ramaiah, D. Ranganathan, H. Razavi, M. Regan, J. T. Rehm, D. B. Rein, G. Remuzzi, K. Richardson, F. P. Rivara, T. Roberts, C. Robinson, F. R. De Leòn, L. Ronfani, R. Room, L. C. Rosenfeld, L. Rushton, R. L. Sacco, S. Saha, U. Sampson, L. Sanchez-Riera, E. Sanman, D. C. Schwebel, J. G. Scott, M. Segui-Gomez, S. Shahraz, D. S. Shepard, H. Shin, R. Shivakoti, D. Singh, G. M. Singh, J. A. Singh, J. Singleton, D. A. Sleet, K. Sliwa, E. Smith, J. L. Smith, N. J. Stapelberg, A. Steer, T. Steiner, W. A. Stolk, L. J. Stovner, C. Sudfeld, S. Syed, G. Tamburlini, M. Tavakkoli, H. R. Taylor, J. A. Taylor, W. J. Taylor, B. Thomas, W. M. Thomson, G. D. Thurston, I. M. Tleyjeh, M. Tonelli, J. A. Towbin, T. Truelsen, M. K. Tsilimbaris, C. Ubeda, E. A. Undurraga, M. J. van der Werf, J. van Os, M. S. Vavilala, N. Venketasubramanian, M. Wang, W. Wang, K. Watt, D. J. Weatherall, M. A. Weinstock, R. Weintraub, M. G. Weisskopf, M. M. Weissman, R. A. White, H. Whiteford, N. Wiebe, S. T. Wiersma, J. D. Wilkinson, H. C. Williams, S. R. Williams, E. Witt, F. Wolfe, A. D. Woolf, S. Wulf, P. H. Yeh, A. K. Zaidi, Z. J. Zheng, D. Zonies, A. D. Lopez, M. A. AlMazroa, Z. A. Memish, A. D. Lopez, Disability-adjusted life years (DALYs) for 291 diseases and injuries in 21 regions, 1990-2010: A systematic analysis for the Global Burden of Disease Study 2010. *Lancet* **380**, 2197–2223 (2012). doi:10.1016/S0140-6736(12)61689-4 Medline

13. L. Liu, H. L. Johnson, S. Cousens, J. Perin, S. Scott, J. E. Lawn, I. Rudan, H. Campbell, R. Cibulskis, M. Li, C. Mathers, R. E. Black, Global, regional, and national causes of child mortality: An updated systematic analysis for 2010 with time trends since 2000. *Lancet* **379**, 2151–2161 (2012). doi:10.1016/S0140-6736(12)60560-1 Medline

14. K. Ward, V. Argyle, M. Meade, L. Nelson, The heritability of preterm delivery. *Obstet. Gynecol.* **106**, 1235–1239 (2005). doi:10.1097/01.AOG.0000189091.35982.85 Medline

15. T. P. York, J. F. Strauss 3rd, M. C. Neale, L. J. Eaves, Estimating fetal and maternal genetic contributions to premature birth from multiparous pregnancy histories of twins using MCMC and maximum-likelihood approaches. *Twin Res. Hum. Genet.* **12**, 333–342 (2009). doi:10.1375/twin.12.4.333 Medline

16. G. Zhang, B. Feenstra, J. Bacelis, X. Liu, L. M. Muglia, J. Juodakis, D. E. Miller, N. Litterman, P.-P. Jiang, L. Russell, D. A. Hinds, Y. Hu, M. T. Weirauch, X. Chen, A. R. Chavan, G. P. Wagner, M. Pavličev, M. C. Nnamani, J. Maziarz, M. K. Karjalainen, M. Rämet, V. Sengpiel, F. Geller, H. A. Boyd, A. Palotie, A. Momany, B. Bedell, K. K. Ryckman, J. M. Huusko, C. R. Forney, L. C. Kottyan, M. Hallman, K. Teramo, E. A. Nohr, G. Davey Smith, M. Melbye, B. Jacobsson, L. J. Muglia, Genetic Associations with Gestational Duration and Spontaneous Preterm Birth. *N. Engl. J. Med.* **377**, 1156–1167 (2017). doi:10.1056/NEJMoa1612665 Medline

17. L. J. Muglia, M. Katz, The enigma of spontaneous preterm birth. *N. Engl. J. Med.* **362**, 529–535 (2010). doi:10.1056/NEJMra0904308 Medline

18. R. Arisoy, M. Yayla, Transvaginal sonographic evaluation of the cervix in asymptomatic singleton pregnancy and management options in short cervix. *J. Pregnancy* **2012**, 201628 (2012). doi:10.1155/2012/201628 Medline

19. W. Koh, W. Pan, C. Gawad, H. C. Fan, G. A. Kerchner, T. Wyss-Coray, Y. J. Blumenfeld, Y. Y. El-Sayed, S. R. Quake, Noninvasive in vivo monitoring of tissue-specific global gene expression in humans. *Proc. Natl. Acad. Sci. U.S.A.* **111**, 7361–7366 (2014). doi:10.1073/pnas.1405528111 Medline

20. R. B. Jaffe, P. A. Lee, A. R. Midgley Jr., Serum gonadotropins before, at the inception of, and following human pregnancy. *J. Clin. Endocrinol. Metab.* **29**, 1281–1283 (1969). doi:10.1210/jcem-29-9-1281 Medline

21. M. Cocquebert, S. Berndt, N. Segond, J. Guibourdenche, P. Murthi, L. Aldaz-Carroll, D. Evain-Brion, T. Fournier, Comparative expression of hCG β-genes in human trophoblast from early and late first-trimester placentas. *Am. J. Physiol. Endocrinol. Metab.* **303**, E950–E958 (2012). doi:10.1152/ajpendo.00087.2012 Medline

22. K. Spencer, N. J. Cowans, F. Molina, K. O. Kagan, K. H. Nicolaides, First-trimester ultrasound and biochemical markers of aneuploidy and the prediction of preterm or early preterm delivery. *Ultrasound Obstet. Gynecol.* **31**, 147–152 (2008). doi:10.1002/uog.5163 Medline

23. R. Romero, S. K. Dey, S. J. Fisher, Preterm labor: One syndrome, many causes. *Science* **345**, 760–765 (2014). doi:10.1126/science.1251816 Medline

24. A. Conde-Agudelo, R. Romero, Vaginal progesterone to prevent preterm birth in pregnant women with a sonographic short cervix: Clinical and public health implications. *Am. J. Obstet. Gynecol.* **214**, 235–242 (2016). doi:10.1016/j.ajog.2015.09.102 Medline

25. J. Davila, M. J. Laws, A. Kannan, Q. Li, R. N. Taylor, M. K. Bagchi, I. C. Bagchi, Rac1 regulates endometrial secretory function to control placental development. *PLOS Genet.* **11**, e1005458 (2015). doi:10.1371/journal.pgen.1005458 Medline

26. S. Hou, S. D. Pauls, P. Liu, A. J. Marshall, The PH domain adaptor protein Bam32/DAPP1 functions in mast cells to restrain FcεRI-induced calcium flux and granule release. *Mol. Immunol.* **48**, 89–97 (2010). doi:10.1016/j.molimm.2010.09.007 Medline

27. Z. Xie, E. C. Chan, K. M. Druey, R4 regulator of G protein signaling (RGS) proteins in inflammation and immunity. *AAPS J.* **18**, 294–304 (2016). doi:10.1208/s12248-015-9847-0 Medline

28. S. Yamamoto-Mizuma, G.-X. Wang, L. L. Liu, K. Schegg, W. J. Hatton, D. Duan, T. L. Horowitz, F. S. Lamb, J. R. Hume, Altered properties of volume-sensitive osmolyte and anion channels (VSOACs) and membrane protein expression in cardiac and smooth muscle myocytes from Clcn3-/- mice. *J. Physiol.* **557**, 439–456 (2004). doi:10.1113/jphysiol.2003.059261 Medline

29. M. J. Berridge, Smooth muscle cell calcium activation mechanisms. *J. Physiol.* **586**, 5047–5061 (2008). doi:10.1113/jphysiol.2008.160440 Medline

30. H. Sasaki, O. Kawano, K. Endo, E. Suzuki, H. Yukiue, Y. Kobayashi, M. Yano, Y. Fujii, Human MOB1 expression in non-small-cell lung cancer. *Clin. Lung Cancer* **8**, 273–276 (2007). doi:10.3816/CLC.2007.n.006 Medline

31. G. R. Saade, K. A. Boggess, S. A. Sullivan, G. R. Markenson, J. D. Iams, D. V. Coonrod, L. M. Pereira, M. S. Esplin, L. M. Cousins, G. K. Lam, M. K. Hoffman, R. D. Severinsen, T. Pugmire, J. S. Flick, A. C. Fox, A. J. Lueth, S. R. Rust, E. Mazzola, C. Hsu, M. T. Dufford, C. L. Bradford, I. E. Ichetovkin, T. C. Fleischer, A. D. Polpitiya, G. C. Critchfield, P. E. Kearney, J. J. Boniface, D. E. Hickok, Development and validation of a spontaneous preterm delivery predictor in asymptomatic women. *Am. J. Obstet. Gynecol.* **214**, 633.e1–633.e24 (2016). doi:10.1016/j.ajog.2016.02.001 Medline

32. D. G. Altman, L. S. Chitty, New charts for ultrasound dating of pregnancy. *Ultrasound Obstet. Gynecol.* **10**, 174–191 (1997). doi:10.1046/j.1469-0705.1997.10030174.x Medline

33. M. Kuhn, Building Predictive Models in *R* Using the caret Package. *J. Stat. Softw.* **28**, (2008). doi:10.18637/jss.v028.i05

34. M. D. Robinson, G. K. Smyth, Small-sample estimation of negative binomial dispersion, with applications to SAGE data. *Biostatistics* **9**, 321–332 (2008). doi:10.1093/biostatistics/kxm030 Medline

35. M. D. Robinson, D. J. McCarthy, G. K. Smyth, edgeR: A Bioconductor package for differential expression analysis of digital gene expression data. *Bioinformatics* **26**, 139–140 (2010). doi:10.1093/bioinformatics/btp616 Medline

36. S. P. Lund, D. Nettleton, D. J. McCarthy, G. K. Smyth, Detecting differential expression in RNA-sequence data using quasi-likelihood with shrunken dispersion estimates. *Stat. Appl. Genet. Mol. Biol.* **11**, 1544-6115 (2012). doi:10.1515/1544-6115.1826 Medline

37. D. J. McCarthy, Y. Chen, G. K. Smyth, Differential expression analysis of multifactor RNA-Seq experiments with respect to biological variation. *Nucleic Acids Res.* **40**, 4288–4297 (2012). doi:10.1093/nar/gks042 Medline

38. M. Waskom *et al.*, mwaskom/seaborn: v0.8.1. *Github* (2017).

39. N. C. Rose, M. T. Mennuti, Maternal serum screening for neural tube defects and fetal chromosome abnormalities. *West. J. Med.* **159**, 312–317 (1993). Medline

40. T. E. Sweeney, W. A. Haynes, F. Vallania, J. P. Ioannidis, P. Khatri, Methods to increase reproducibility in differential gene expression via meta-analysis. *Nucleic Acids Res.* **45**, e1 (2017). doi:10.1093/nar/gkw797 Medline
